# Views of the Swiss public towards gene editing

**DOI:** 10.1371/journal.pone.0334305

**Published:** 2026-05-21

**Authors:** Kelly E. Ormond, Sara Kijewski, Naomi Wyler, Agata Ferretti, Eirini Petrou, Claudia Reichmuth, Effy Vayena

**Affiliations:** 1 Department of Health Sciences and Technology, Health Ethics and Policy Lab. ETH Zürich, Zürich, Switzerland; 2 Medizinische Fakultät, Universität Zürich, Zürich, Switzerland; University of Basel Institute for Biomedical Ethics: Universitat Basel Institut fur Bio- und Medizinethik, SWITZERLAND

## Abstract

There is little country-specific data about how the general public views gene editing therapies. In Autumn 2023 we randomly surveyed the Swiss public, using the Federal Register and stratifying by language region (German, French, Italian), gender and age. We present a representative sample of 3855 responses, including >4000 open-ended comments. When presented with 7 therapeutic options for somatic gene editing, 7% disagreed with all therapeutic options, and 35% supported them all. Most agreed with using somatic gene editing to cure life-threatening (76%) and debilitating diseases (70%); support declined as severity decreased or with later onset. Few supported somatic gene editing to enhance physical (6%) or cognitive (9%) abilities. In all scenarios, people were less likely to agree with gene editing of embryos. Notably, all therapeutic gene editing attitudes clustered, regardless of somatic vs. germline differentiations. Factor analysis also demonstrated two clusters for “support” and “caution” towards gene editing, and multivariate analysis demonstrated relationships with age, gender, religion and knowledge. When asked what influenced their views, the most endorsed reasons for feeling positively were ‘views towards what it means to have a good life’ (59.8%) and ‘views about illness and suffering’ (58.5%). Most selected “neutral” to describe religion’s influence (68.9%) despite findings that those who endorsed high religiosity were less supportive and more cautious towards gene editing. Conclusions: Uncovering systematic differences in the attitudes towards specific therapies and the values shaping them underscores the importance of including peoples’ voices in policy decisions in a country-specific manner.

## Introduction

Discussions of public views towards gene therapies and gene editing have been published since the early 1990s, but their frequency has increased in the past decade as CRISPR-Cas9 and newer therapies have made the option more technologically feasible in the near term. There is a range of papers that provide insight to the public’s views, many reviewed by Delhove et al (2020) [1], but they are challenged by covering only germline gene editing, [2–5] using different methodologies (survey versus public engagement events, for example), or phrasing questions in different (non-validated) manners. Additionally, several were published in 2016–2017, [6, 7] when the technology was still very hypothetical.

Nevertheless, some data exists describing views of the general public towards gene editing from the US, Canada, UK, EU, Netherlands, South Africa, Japan, China and Australia. Papers assessing the general public generally show greater support for somatic gene editing (SGE) than for germline gene editing (GGE) [1, 7–9]. Only two large public studies show non-significant differences between these views [6, 10]. Studies also generally agree that support increases with disease severity [2, 4, 6, 8, 11–13] and decreases when considering enhancement (for example of physical or cognitive features) [4–7]. Importantly, some significant part of the population often disagrees with any use –for example in Japan up to 30% of the general public were not supportive [8, 14].

Finally, factors that seem to influence public views have also been examined, including as part of the Delhove review. Most studies that examined religiosity found that those who self-describe as more religious have lower support for gene editing [1, 5, 6, 10, 12, 13], particularly for GGE [3]. When age played a role, younger individuals were more supportive [1, 5]. Education level was associated with higher support for gene editing [1, 12, 13, 15]. When gender differences were found, men were more supportive [1, 5, 6, 8, 10, 16]. Personal or family history of genetic disease showed mixed findings: some studies suggested this did not influence views towards gene editing [1, 13], but studies of patient and family stakeholders identified specifically around genetic disease groups showed higher interest [4, 17–21].

Several authors comment that country-specific data will be useful to gather given the important differences noted thus far. Country-specific data can guide policy creation in a way that recognizes important values and cultural differences that may not otherwise appear. For example, Switzerland is a small multilingual and multicultural country with high literacy rates [22]. We recently collected data on how scientific and medical professionals in Switzerland view gene editing [23], but what is not known is how the Swiss public thinks about the potential for gene editing. As such, we constructed the current study to assess the attitudes of the Swiss public towards gene editing, including how these attitudes compare to the already assessed public views.

## Materials and methods

We conducted an online cross-sectional survey using Qualtrics to learn about the opinions, concerns and expectations of Swiss residents towards gene editing.

### Ethics approval statement

The survey was approved by the ETH Zürich Ethics Commission (EK 2022-N-84). Participants reviewed the written informed consent materials before completing the survey, which included the statement “By participating in the survey, you are giving your informed consent to participate in this research study.”

### Subjects

Potential participants were 18–72 years old and lived in Switzerland. In order to best represent the general public of Switzerland, the Swiss Federal Statistical Office (FSO) provided a stratified random sample across sex, age (4 groups, between 18 and 64+), and three main language regions (German, French, Italian) in Switzerland. Since a majority (62%) of the Swiss population speaks primarily German, and only 23% and 8% speak French and Italian, respectively, the minority languages were oversampled (2:1:1 for German, French, Italian) in order to maximize responses from these populations. Anticipating an approximately 30% response rate, the target population included 14,825 individuals (6977 German speakers, 4013 French speakers, and 3835 Italian speakers).

### Questionnaire development

The survey (Supplemental Methods, in English) was based on an unpublished survey that was used by the Australian Citizen Jury research team (personal communication, S Niemeyer). Several questions were added after interviews with Swiss experts to obtain their views on gene editing and expectations for areas where the Swiss public might differ uniquely [23]. The survey was divided into four parts. In the first part questions were asked about the participants’ background such as canton of residency, gender, age, education level, religious beliefs and political attitude. In the second part two questions about the awareness and pre-knowledge about gene editing were asked. Then, a short explanation about gene editing in simple language was given to all participants. In the third part, views on human gene editing (18 questions) and its potential usages (15 questions) were investigated. We used a Likert Scale either ranging from 1–5 or 1–7, where 1 = strongly disagree and 5 or 7 = strongly agree. The fourth part contained questions about the attitudes towards the regulation of human gene editing. The operationalization of these variables is described in the Supplemental Methods (S1 File).

The survey was created in English, and then translated to German, French and Italian using DeepL (an artificial intelligence translator) and then error checked and edited for fluency by at least two team members for whom the language was their mother tongue. Non-English versions of the survey are available on request.

### Recruitment

Recruitment mailings were sent between September 7 and October 5 2023. Potential participants were mailed a letter in their preferred language of correspondence that briefly described the study and gave instructions to access the survey, either online or to request a paper version. A secured individualized code that links the data to the sociodemographic data provided by FSO was given in order to be able to anonymously track the individual responses. Two reminder notifications were sent to non-responders at two and four weeks. The data was stored safely in an encrypted form at ETH.

The survey was administered over Qualtrics (Provo, UT; version accessed between June 2023 and June 2024) and participants could toggle between four different languages (English, German, French, Italian) while completing the survey if desired. Forty paper surveys were requested; these were mailed with the secured code and entered by hand by a single researcher. The final sample contained 3855 respondents who completed attitudinal questions beyond demographics.

### Statistical analysis

We analyzed the data using the software Stata (version Stata/SE 17.0 for Mac, StataCorp, College Station) and produced the coefficient plots using RStudio (version 2025.09.2 + 418). To reduce sampling error and account for non-response bias, we applied post-stratification survey weights in the data analysis that were calculated based on gender, age and language region. To examine variation in individual views on gene editing, the data was examined using ordinary least squares-regression analysis. For this analysis, we created factor variables estimated based on maximum-likelihood factor analysis of a polychoric correlation matrix, given the ordinal nature of the variables (the operationalization of variables is shown in Supplemental Table 1). To allow the factor variables to correlate, we applied promax rotation. Our analysis included age, gender, level of education, religiosity, whether the respondent or a family member has an inherited or genetic condition, experience in medical or related fields, political participation, level of knowledge on gene editing, language and region. All predictors were included a priori based on their use in a prior study (e.g., the Australian study from which the survey was based or a prior Swiss-wide study on personalized medicine [24] and theoretical relevance. No stepwise or data-driven variable selection was employed. Multicollinearity was assessed using variance inflation factors (VIF); all values were below 4.95, indicating no cause for concern. Further, we included data provided on household size, marital status and nationality acquired from the FSO. The statistical analysis was conducted based on complete cases, using listwise deletion for missing data.

### Qualitative analysis

The survey contained 5 open-ended questions that resulted in 4095 comments, which were qualitatively analyzed using thematic analysis [25]. In order to get an overview of the mentioned themes, select fluent members of the research team (KO, NW, CR, EP, AF) reviewed comments in the original languages to inductively create a draft code book and definitions. The codebook was then tested using 30 English comments (since all researchers were fluent in English). After codebook revisions, a second training round with 10 English comments was conducted. The final code book was then used to analyze the data in NVivo (version 20). To maximize rigor, 20% of the comments were co-coded by two members. The codes were thematically analyzed to understand the main themes and to assist in interpreting the quantitative study results.

## Results

### Sample characteristics

298 survey invitations were returned due to incorrect addresses and 23 persons responded to refuse survey participation. Of the remaining potential participants, 4171 surveys were started online (response rate = 28.6%), and 3855 surveys were included in the final sample, with 3429 included in the main regression analysis. Table 1 describes the participants’ demographics in the final sample (n = 3855). 82% of participants were Swiss, and the population was approximately 50% women with a mean age 48 ± 16 (SD); 54% were married and 10% self-report they are very religious. All language regions were represented, with an over-representation of participants from the Italian speaking region (Ticino) (33% of responses vs. 4% of population). Twenty-seven percent of the participants had a university degree, and 10% worked in a related field of biology or medicine. Approximately 20% reported an inherited disease in their family.

**Table 1:** Participant Demographics.

Variable	Number of responses in final sample (N = 3855)	Unweighted percentage (%)	Weighted percentage (%)
**Language of survey completion**			
German	1706	44.3	66.7
English	163	4.2	5.4
Italian	1210	31.4	5.1
French	776	20.1	22.8
Total	3855	100	100
**Gender**			
Male	1847	48.0	48.7
Female	1942	50.4	49.7
Non-binary/fluid	15	0.4	0.4
Prefer not to say	47	1.2	1.2
Total	3851	100	100
**Age**			
18-24	468	12.2	8.6
25-35	652	17.0	14.8
35-44	586	15.2	18.3
45-54	708	18.4	17.3
55-64	842	21.9	17.6
65+	591	15.4	23.4
Total	3847	100	100
**Nationality**			
Swiss	3088	80.0	81.8
Other	766	20.0	18.2
Total	3854	100	100
**Household size**			
1 person	574	14.9	15.2
2 people	1310	33.9	38.5
3-5 people	1905	49.3	44.6
6 or more people	74	1.9	1.7
Total	3855	100	100
**Marital status**			
Single	1505	39.0	33.7
Married	1932	50.1	54.1
Widowed	65	1.7	2.0
Divorced	344	8.9	10.0
Registered partnership	7	0.2	0.2
Dissolved registered partnership	2	0.1	0.1
Total	3855	100	100
**Educational level**			
Up to Secondary education or Other	2756	71.8	73.3
Tertiary education (Bachelor's degree or higher)	1084	28.2	26.7
Total	3840	100	100
**Work experience in a related field (Medicine, Biology, Genetics etc.)**			
Yes	389	10.1	9.9
No	3474	89.9	90.1
Total	3855	100	100
**Self-reported awareness of gene editing**			
Never heard of gene editing	406	10.6	6.2
Aware,limited knowledge	2500	65.1	68.3
Knowledgeable	755	19.7	21.1
Unsure/don’t know	179	4.7	4.4
Total	3840	100	100
**Self-reported level of religiosity**			
Very much	361	9.4	9.6
Somewhat	1713	44.8	43.4
Not at all	1751	45.8	47.0
Total	3825	100	100
**Voted in last election**			
Yes	2241	58.4	60.4
No	721	18.8	18.5
No, not eligible to vote	651	17.0	16.0
Prefer not to say	227	5.9	5.2
Total	3840	100	100
**Genetic/inherited disease in family**			
Yes	737	19.3	19.6
No	2182	57.1	56.8
Unsure/don't know	821	21.5	21.8
Prefer not to say	84	2.2	1.8
Total	3824	100	100

With regard to awareness of gene editing, most consider themselves knowledgeable (21%) or aware with limited knowledge (68%); only 6% reported having never heard of human gene editing before the survey. The level of knowledge about gene editing among the respondents is fairly high: 50% of the respondents respond correctly to at least 4 of the 5 questions measuring factual knowledge about genetics and gene editing. At the conclusion of the survey, most participants reported feeling very (29%) or somewhat confident (48%) in expressing their views about gene-editing on the survey.

### Views on gene editing

Table 2, 3 provide frequency responses to questions assessing general attitudes towards gene editing and to what degree respondents supported or disagreed with specific applications of SGE and GGE. There was a wide range of opinion on all the attitudinal questions, with many participants responding in a neutral manner. A sub-analysis of the neutral responses for the 18 items described in Table 2 revealed that 71% of the respondents report five or less neutral responses, suggesting that most respondents do have an opinion on gene editing. There is, however, a minority of 7% (n = 239) that report 10 or more neutral responses, and there is a small spike at “only neutral responses”, containing 3% of the respondents. To examine whether neutral responses reflected systematic avoidance or uncertainty, we identified high neutral responders, defined as respondents selecting the neutral option on more than 10 of 18 items. Chi-square tests showed that high neutral responding was significantly associated with lower education (9.0% vs. 2.1% among tertiary educated respondents, χ²(1)=52.6, p < 0.001), Italian language region (10.7% vs. 4.0% among German speakers, χ²(3)=43.6, p < 0.001), and higher religiosity (9.3% among very religious vs. 5.7% among non-religious respondents, χ²(2)=8.4, p = 0.015). Age and gender were not significantly associated with high neutral responding (p = 0.594 and p = 0.970, respectively). The concentration of neutral responses among those with lower education and stronger religiosity could signify genuine uncertainty related to an unfamiliar or ethically complex technology, rather than systematic opinion avoidance. Language region differences may reflect differential familiarity with gene editing across Swiss linguistic communities or cultural variation in response styles, yet this warrants deeper analysis. When examining the relationship between the frequency of neutral responses, support of and caution towards gene editing, we find that neutral responses are negatively related to both support (r = −0.413) and rejection (r = −0.413), with both relationships significant at the 1%-level. Support and rejection are also associated (−0.490, at the 1%-level). The similarly strong association between a high frequency of neutral responses and both support and rejection may suggest that respondents are genuinely uncertain about their choice and not “soft” supporters or opposers.

**Table 2:** Attitudes towards gene editing (percentages in weighted sample).

	Strongly disagree			Neutral	Strongly agree		
	1	2	3	4	5	6	7
Parents have a right to edit the genes of their children before they are born (n = 3792)	34.3	14.3	11.4	22.4	8.1	4.7	4.7
I'm concerned that gene editing will lead to a reduction in genetic diversity (n = 3799)	11.5	10.8	8.0	27.0	16.4	12.9	13.5
I'm concerned that gene editing will be used as a quick techno-fix without actually dealing with real problems (n = 3799)	9.9	10.9	8.0	20.8	16.8	15.2	16.4
Editing genes that will be inherited is problematic because it means making decisions for future people who don't exist yet (n = 3800)	8.1	8.1	6.3	19.3	16.9	15.2	26.1
My cultural beliefs make me cautious about gene editing (n = 3802)	21.9	11.3	6.7	21.9	11.0	10.2	17.0
When you change someone’s genes you are fundamentally changing who they are (n = 3792)	15.3	14.8	10.5	22.6	14.1	9.6	13.2
I worry that something will go wrong with gene technology (n = 3801)	5.2	7.0	7.4	20.0	18.9	16.4	25.1
Gene editing technologies give us power over human life itself (n = 3790)	9.0	7.5	7.4	20.3	19.1	18.4	18.3
We find strength when we face illness and adversity. It makes us who we are (n = 3793)	10.7	9.9	7.4	25.9	18.3	13.0	14.7
If gene editing is proven to be safe, then I see no reason to oppose it (n = 3765)	14.0	10.2	7.7	19.6	16.8	14.2	17.5
I would consider using gene editing for my children to give them an advantage in life, so long as it’s safe (n = 3750)	26.7	11.7	7.6	21.7	13.8	9.3	9.2
I would be OK with having my own genes edited (n = 3754)	31.2	11.6	8.0	20.6	12.1	7.9	8.7
Each individual has a right to decide for themselves whether to undergo gene editing, as long as the changes cannot be passed on to future generations (n = 3761)	6.9	5.1	4.8	16.6	14.4	17.4	34.8
If we are able to safely perform gene editing, we should make it available (n = 3756)	13.2	7.2	7.9	21.2	20.1	14.1	16.4
Gene editing should be available only if there are no other treatment options available (n = 3754)	7.7	7.1	6.2	20.1	15.8	19.4	23.8
If gene editing is available in other countries it should be available in Switzerland (n = 3760)	20.4	9.7	8.4	26.5	12.6	10.3	12.2
Gene editing is eugenics (n = 3632)	11.9	5.5	4.6	51.8	10.7	7.5	7.9
Gene editing will increase the inequities that already exist for people with rare disease and disability (n = 3744)	17.1	13.8	10.0	32.5	10.5	7.7	8.5

**Table 3:** Agreement with various forms of gene editing (percentages in weighted sample).

VIEWS TOWARDS SOMATIC GENE EDITING (THERAPEUTIC AND ENHANCEMENT)	1 Strongly disagree	2 Disagree	3 Neutral	4 Agree	5 Strongly agree
Editing the cells of children or adults to cure a life-threatening disease(n = 3742)	5.8	4.3	13.5	35.7	40.6
Editing the cells of children or adults to cure a debilitating disease (n = 3721)	6.6	6.3	19.0	37.8	30.4
Editing the cells of children or adults to cure a disease that led to minor physical impairments (n = 3718)	11.4	14.5	29.5	28.3	16.3
Editing the cells of children or adults to cure a disease that led to significant learning impairments (for example, a person could not live independently) and also some moderate physical impairments (n = 3716)	9.5	8.7	21.8	33.6	26.5
Editing the cells of children or adults to cure a disease that led to significant learning impairments but without any other medical impacts (n = 3713)	10.1	9.7	24.3	31.5	24.4
Editing the cells of children or adults to cure a disease that started only in adulthood (for example, cancer or dementia) (n = 3713)	8.7	7.8	19.9	31.3	32.3
Editing the cells of children or adults to cure a disease that led to a sensory impairment, such as vision or hearing loss from birth (n = 3705)	8.7	6.3	18.7	33.3	33.0
Editing the cells of children or adults to alter physical abilities—such as strength or sporting ability (n = 3714)	58.8	22.8	13.2	3.3	2.0
Editing the cells of children or adults to alter cognitive abilities –such as memory or intelligence (n = 3702)	53.5	22.0	16.9	5.3	2.6
VIEWS TOWARDS HUMAN GERMLINE GENE EDITING (THERAPEUTIC AND ENHANCEMENT)	1 Strongly disagree	2 Disagree	3 Neutral	4 Agree	5 Strongly agree
Editing the cells of embryos to prevent a life-threatening disease (n = 3695)	15.5	8.1	19.8	27.8	28.9
Editing the cells of embryos to prevent a debilitating disease (n = 3692)	17.8	12.3	25.1	26.4	18.4
Editing the cells of embryos to alter physical abilities—such as strength or sporting ability (n = 3696)	63.2	20.0	12.6	2.5	1.6
Editing the cells of embryos to alter cognitive abilities—such as memory or intelligence (n = 3692)	58.5	20.0	14.8	4.1	2.6
OTHER RELATED VIEWS	1 Strongly disagree	2 Disagree	3 Neutral	4 Agree	5 Strongly agree
Editing the cells of plants and animals used in food production (n = 3686)	29.1	17.7	27.3	16.7	9.2
Editing human embryos in genome editing research (n = 3671)	34.4	16.5	30.3	12.2	6.7

Approximately half (48%) of participants felt that if gene editing was safe it should be offered; 20% were neutral on this point. Fifty-nine percent support its availability if no other treatment options exist. Thirty two percent would consider it for their children and 29% would consider it for themselves. Respondents generally endorsed statements that each individual should have the right to make decisions about gene editing as long as the changes were not passed on (67%), and that making decisions for future generations is problematic (58%); 60% did not feel that parents had the right to edit their children’s genes before birth (T2). If gene editing is available in other countries, 35% felt it should also be available in Switzerland.

When asked about which of 7 possible hypothetical uses of SGE they would support (T3), 7% disagreed with all therapeutic SGE options, 7% were neutral on all options, and 47% supported either 6 (14%) or all 7 options (33%). A majority of the Swiss public agreed with SGE for life threatening disease (76%) or debilitating disease (68%), and this agreement lessened for germline treatment of the same (57% and 45% respectively) or for more minor physical, learning or sensory impairments. Many hoped gene editing would treat or cure many conditions –the most named conditions were cancer, dementia, psychiatric illness, and conditions described as ‘severe’, ‘causing suffering’, ‘uncureable’ or those without other treatments.

*Improving human life, reducing physical and mental suffering. (original language French)*

*I think it would be good to combat life-threatening diseases such as cancer. Many people are affected by it and suffer from it and it would be revolutionary if it worked. (original language German)*

Participants were asked to what degree they endorsed potential concerns. A moderate number agreed with concerns about safety (‘something going wrong’: 60%), reduction in genetic diversity (43%), fundamentally changing who a person is (37%), or that gene editing would exacerbate already existing inequalities (27%) or was eugenic (26%). Open ended comments elaborated, including many comments that treatments are not ‘natural’.

*Human beings are imperfect and so they must remain. Unique and special. (original language Italian)*

*I think people shouldn’t interfere with nature. One day we will no longer be humans but creatures. (original language German)*

*I don't think it's up to our generation to decide which genes will be better for future generations. (original language French)*

Others suggested a fear of commercialization or lack of regulation:

*Do we really want to give corporations even greater control over humanity? The result will be accelerating global inequalities. (original language French)*

*I see a danger in the inability to really control what goes on in laboratories in countries under authoritarian regimes where it could get out of control and generate an irretrievable drift of humanity. (original language Italian)*

Gene editing for enhancement was supported by less than 10% of respondents (T2) in either SGE or GGE. Open ended comments elaborated, frequently likening such enhancement gene editing to ‘playing god’:

*I am against playing God, not everything that is possible must be allowed. I am against parents being able to put together their children like from a catalog. (original language German)*

*Gene editing may only be used to cure or prevent serious diseases, never to change characteristics such as strength or intelligence. Keyword “superhuman”. (original language German)*

When participants were asked to endorse a list of factors that may have influenced their views towards gene editing, the most endorsed factors (nearly always citing a positive influence) included: what it means to ‘have a good life’ (57%), views towards illness and suffering (53%), views towards science and biology (50%), and what it means to be a parent and have children (49%). Most (62%) felt their personal views towards religion had a neutral influence on views towards gene editing. Open ended comments confirmed many of these factors.

*I believe that humans are generally happier when they are healthy and free from diseases. Editing genes is a tool that has the potential to work towards that goal. (original language English)*

*If it's possible to modify to make life easier for someone in difficulty, or to prevent a complicated life, then I'm all for it (original language French)*

*God has created the world and all life in it. Life is precious and experiences are a part of life. Pain and suffering are inescapable and help us to grow as individuals and as a community. How we learn and use these experiences is up to each person. (original language English)*

### Correlates of views on gene editing

To understand which individual factors associate witht the attitudes described above, we conducted multivariate analyses examining individual determinants of both broader attitudes toward gene editing and support for specific applications. Supplemental Table 2 describes the demographics of the sample used for this regression analysis (N = 3429).

#### Correlates of support and caution towards human gene editing.

**Fig 1:**
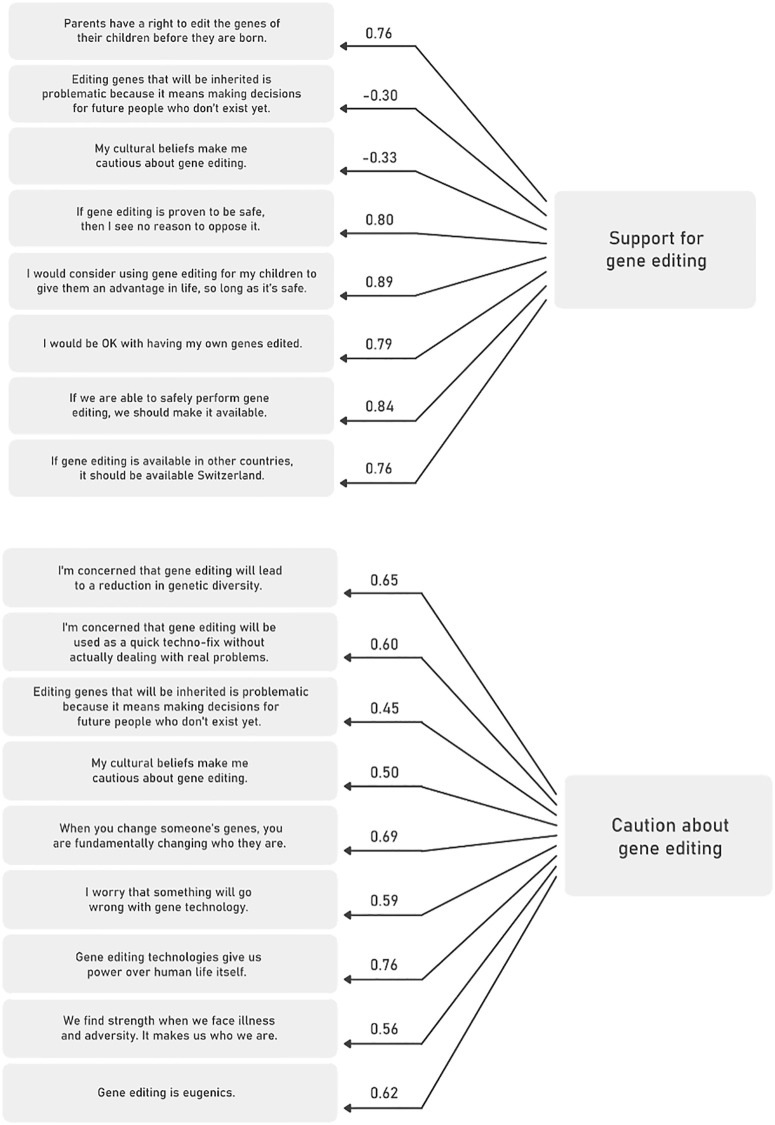
a: Factor structure and item loadings for support for gene editing, n = 3505. b: Factor structure and item loadings for caution about gene editing, n = 3505.

For views on gene editing under specific circumstances, the factor analysis uncovered two latent concepts in the data: one variable indicating support of gene editing, and another one expressing caution about gene editing (see Figs 1a-b). There is a moderate correlation between the two, which is highly statistically significant (−.46, p = 0.001), indicating that respondents who highly support gene editing express moderately low caution.

**Fig 2:**
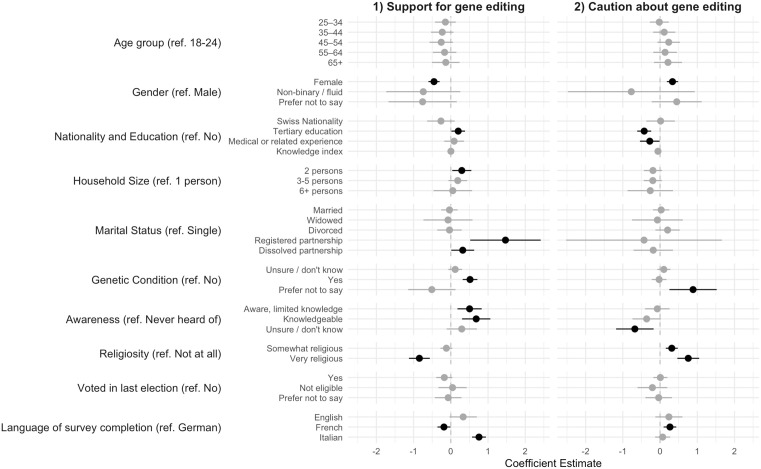
Correlates of Support and Caution Toward Gene Editing, n = 3429. *Note:* Coefficients and 95% confidence intervals from weighted linear regression models. Statistically significant coefficients are bold.

Fig 2 presents the relationships between various individual variables and these support or caution factors (weighted ordinary least squares regression analysis, weighted and unweighted results are substantively similar (see Supplementary Table 3). Several demographic patterns emerge consistently across both factors. Gender is strongly associated with views on gene editing, with female respondents showing significantly lower support and also more caution about gene editing (both p = 0.000) compared to male respondents. Education also has a consistent effect, with tertiary education being associated with higher support (p = 0.031) and lower caution (p = 0.000) compared to secondary or other levels of education.

Religiosity appears to have a more nuanced relationship with both attitudes. Very religious individuals showed significantly lower support for gene editing compared to those respondents who report they are not religious at all (p = 0.000). For caution, we find that both moderate and strong religiosity is linked to higher levels of caution (both p = 0.000), with being very religious having the strongest effect.

Personal relevance factors were especially important for support. Individuals who indicated that they or their family members have an inherited genetic condition showed higher support (p = 0.000) compared to those who did not report any of these conditions, as did those who self-reported awareness or knowledge of gene editing (p = 0.002 and p = 0.000 respectively). Experience in a medically or related field is linked to a lower level of caution (p < 0.05). Regional differences were also evident, with Italian-speakers more and French-speakers less supportive of gene editing than German-speakers (p = 0.000 and p = 0.039 respectively).

To better understand whether a neutral position reflects a moderate position or limited engagement with the topic, we conducted a sub-analysis of the link of a high number of neutral responses and knowledge about gene editing. We found that this relationship between neutrals and knowledge is moderately negative and significant at the 1%-level (r = −0.40). Similar, but weaker, associations are found with having experience from medical fields and tertiary education. Both support and rejection are positively related to knowledge (r = 0.27 and r = 0.16, respectively, both statistically significant at the 1%-level).

### Correlates of views towards human gene editing uses and applications

**Fig 3:**
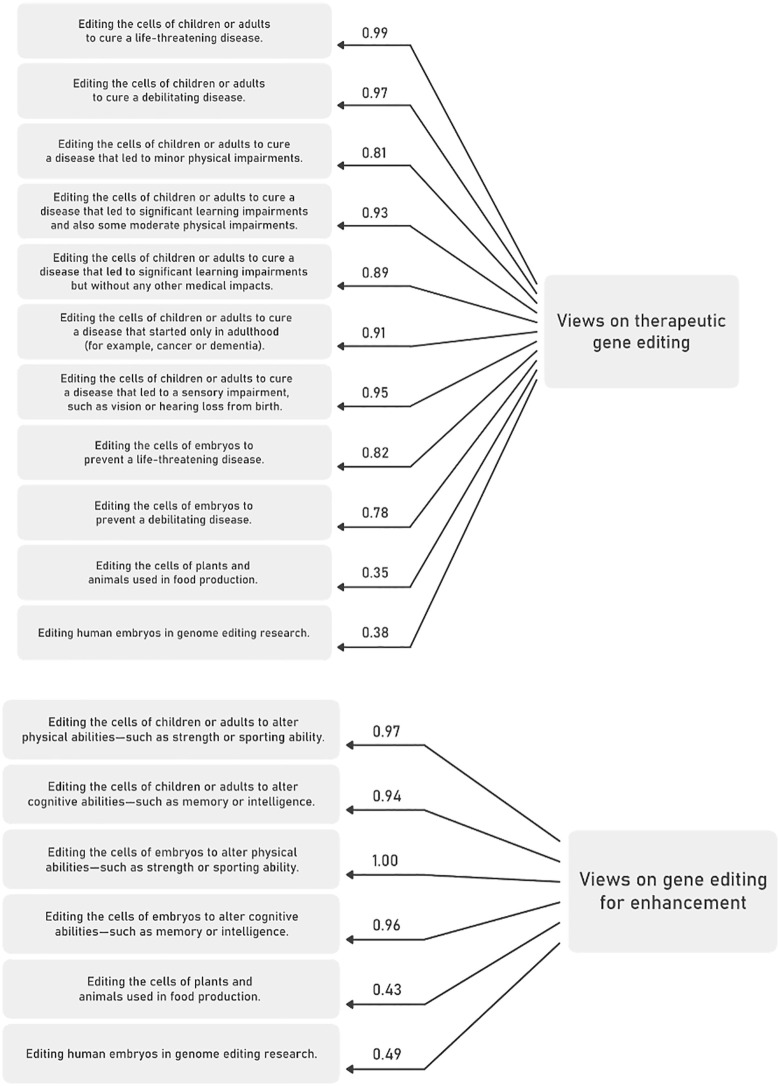
a: Factor structure and item loadings for views on therapeutic gene editing, n = 3587. b: Factor structure and item loadings for views on gene editing for enhancement, n = 3587.

In a next step, we examined the association of variables with support for specific uses of gene editing. Through factor analysis, we identified two measures (see Fig 3a-b) for views on potential uses of gene editing: views on therapeutic gene editing and views on enhancement applications. The factor analysis suggested that respondents did not distinguish between SGE and GGE in either application. For example, items that refer to editing cells of human embryos load on the same factor (factor loadings 0.82 and 0.78) as items that refer to editing cells of children and adults to cure a debilitating disease (factor loading 0.97).

**Fig 4:**
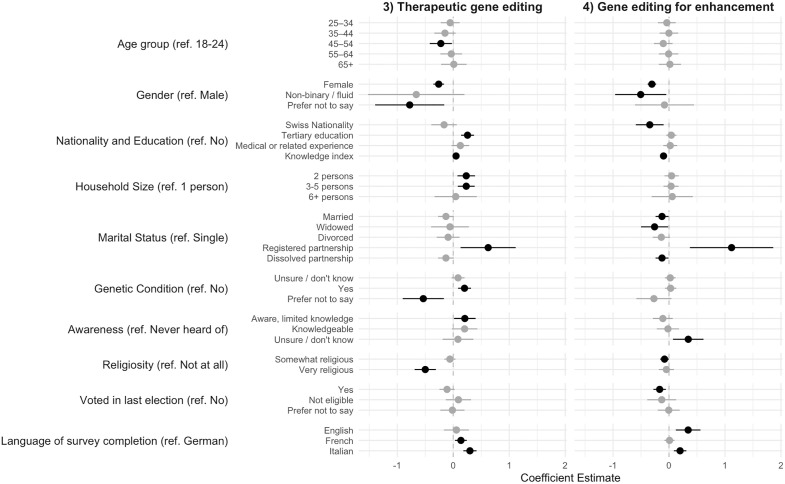
Correlates of Views on Therapeutic Gene Editing and Gene Editing for Enhancement, n = 3490. *Note:* Coefficients and 95% confidence intervals from weighted linear regression models. Statistically significant coefficients in bold.

Fig 4 displays the results of the linear regression analysis (see full weighted and unweighted results in Table 4 in the supplementary materials; again, weighting did not change the results substantially). Consistent patterns across applications mirrored those for support and caution towards gene editing. Gender remains statistically associated, with female respondents reporting a moderately lower level of agreement for both therapeutic (p = 0.000) and enhancement (p = 0.000) uses. Similarly, Italian-language was positively related to both types of gene editing (p = 0.000 and p = 0.001 respectively).

Knowledge showed contrasting effects across the two types of uses: Higher knowledge on gene editing was positively related to support for therapeutic gene editing (p = 0.020) but negatively for enhancement (p = 0.000), indicating that those more knowledgeable were skeptical of enhancement.

For therapeutic gene editing specifically, several additional associationss emerged. The age group between 45 and 54 was less supportive than the 18–24 year olds (p = 0.028). Larger household sizes (2, or 3–5 household members) (p = .004 and p = .003), tertiary education (p = 0.000), personal relevance (p = 0.001) and awareness (p = 0.034), as well as French-language completion of the survey (p = 0.011), were all related to higher support for therapeutic gene editing. Very religious respondents showed lower support (p = 0.000).

For enhancement use, the associations differed notably. Our analyses found that marriage (p = 0.032) or widowhood (p = .037), political engagement, measured as voting in the last election (p = 0.004), and Swiss Nationality (p = 0.007) were all negatively related to views on gene editing for enhancement. Being female (p = 0.000) or non-binary/fluid gender identity (p = .030) were also related to a lower level of agreement. English- and Italian-speaking respondents showed higher support for uses of gene editing for enhancement than German-speaking respondents (p = 0.002 and p = 0.001 respectively).

### How should Swiss policies be formulated about gene editing?

We asked participants about whose voices should be heard in order to formulate policies on gene editing. Groups that were seen as completely necessary to the discussion included Swiss experts (scientists, doctors, lawyers and bioethicists; 45%), people with inherited diseases (32%) and ordinary people (30%). If a citizen jury would be held, it was most important to survey respondents that it includes presentations from experts on gene editing (47%) and from people who have lived experiences of the conditions that might potentially be treated (45%). Statements or input from international organizations (e.g., UN, WHO, OECD or EC) were seen as less necessary than hearing specifically from these Swiss groups. Finally, many of the open-ended comments at the end of the survey expressed the desire for the Swiss public to be further educated and engaged in discussions about gene editing.

*Since the political debate hasn't yet been conducted at a societal level, the individual positions are still unclear and lacking in differentiation. (original language German)*

## Discussion

Our study documents a representative survey of Swiss residents in regards to their support and reasonings towards gene editing in Autumn 2023. This survey finds that Swiss people are moderately supportive of gene editing. We found slightly higher support of SGE than GGE, highest support for treating more serious conditions, and significantly less support for enhancement gene editing at any stage. Support was higher in those who had a family member with an inherited condition, and our data strongly supports a role of knowledge and awareness in views on gene editing for therapeutic applications. Those who were more cautious towards gene editing were more likely to be women or to have stronger self-reported religiosity. Only 7% hypothetically rejected all forms of therapeutic SGE. Finally, it was interesting that many of our questions showed approximately 20% of respondents neutral to each inquiry, and that there was a negative relationship between knowledge and neutral responses. This may indicate that knowledge is associated with the formation of definitive opinion, whereas limited engagement and knowledge may correlate with a neutral position.

When we compare the current study data with previously published papers, we note many consistencies with the existing literature. First, the moderately high acceptance of SGE approaches is consistent with the expectations we had based on our prior study of Swiss professionals [23]. Additionally, the Swiss report moderate decreases in support (15–20% less) for treating similar indications when performed in embryos as compared to in children or adults. This differentiation between somatic and germline is consistent with prior studies [1, 7–9]. However, the factor analysis loaded both questions referring to both editing cells of human embryos and cells of children or adults onto the same factor. This suggests that while absolute support levels are differing, support for somatic and germline editing for both therapeutic and enhancement are related to the same underlying attitudes. This may reflect that attitudes are linked to the aims of gene editing and whether it is used for therapeutic or enhancement purposes, or that there is limited public awareness of the different implications of these two types of gene editing. We also noted that only 19% of participants supported gene editing research on embryos (Table 3), while approximately half voiced support for various forms of therapeutic GGE. This finding is interesting in several ways – Swiss law does not allow embryo research and historically Swiss have been slower to approve assisted reproductive technologies (for example IVF/PGD). The current data suggests that the Swiss public is more receptive to the notion of GGE than Swiss experts were [23], but not to the degree that a referendum to change current legal restrictions would be successful in the near future. Our data did not allow us to clearly determine if the Swiss public was truly more accepting of GGE (for example because they valued stopping transmission of a genetic condition to future generations) or simply did not understand important factors about the technology. Certainly there were many open ended comments expressing that respondents felt their understanding was limited. We also note that study participants generally endorsed statements that supported the rights of children and future generations to decide for themselves about gene editing (Table 2), which is consistent with a European view towards the UN Convention on the Rights of the Child [26], and specifically the ‘right to participate’.

It was not surprising to find that support for gene editing increases with disease severity and decreases when considering enhancement as these things have been consistently reported in prior literature [2, 4, 6, 8, 11–13] [4–7]. Our study also found that respondents who indicate that they or their family members have an inherited or genetic condition express significantly higher levels of support for human gene editing than respondents reporting not to have any of these conditions. This finding is interesting as most studies of the general public suggested this did not influence views towards gene editing [1], but studies of patient and family stakeholders identified specifically around genetic disease groups showed higher interest [4, 17–21].

Our study results were also consistent with prior literature in showing that women and self-reported very religious individuals show lower support of gene editing [1, 5, 6, 8, 10, 12, 13, 16], particularly in regards to GGE [3]. We note that in our prior study with Swiss experts [23], it was predicted that religion would not be a major factor for Swiss persons in their attitudes towards gene editing, and this was also consistent, since in fact most respondents to this survey felt that religion was a neutral factor in their views. We also found that higher education (generally) and knowledge about gene editing are also positively related to higher support and lower caution towards at least therapeutic gene editing [1, 6, 12, 16].

Finally, our study showed differences in acceptance of various forms of gene editing across the language regions of Switzerland. This reinforces our belief that even within a single country there will be local cultural factors that influence how people view gene editing. It is perhaps a uniquely Swiss finding how many people responded neutrally to the questions (e.g., in Tables 2 and 3). There are several possibilities that could explain this, including that some respondents were simply unsure about how best to answer the questions or, as our sub-analysis suggests, were lacking relevant knowledge, leading them to select a more neutral response. Some of the neutral responses may also reflect the Swiss governmental approaches of direct (and usually consensus based) democracy, perhaps indicating lack of a strong feeling towards either option and willingness to compromise.

### Limitations

Several important methodologic features must be considered when interpreting our results. First, our response rate was slightly lower than 30%, although the population is generally representative of the Swiss population with the exception that it over-represents the Italian speaking region (Ticino) (33% of responses, versus 4% of the Swiss population in the final sample), and an under-represents the German speaking region (46% vs. 62%). While our analysis standardized for these variations, which increases the chance that the impact on our results and interpretation is minimal, it remains possible that some response bias exists that would limit generalization to the study population rather than the Swiss population. It is certainly possible that those with stronger opinions (either for or against) were more likely to respond to the survey. Second, since many tests were performed without Bonferroni correction, it is possible that some positive findings reflect Type 1 errors. Third, missing data and listwise deletion excluded several respondents from the regression analyses. Further checks indicated that missing values were mostly concentrated on the dependent variables. The support and caution factors, for example, had 350 missing values (9.1%), and the views on therapeutic and enhancement uses had 277 missing values (7.2%). Those that skipped questions included in these variables tended to be older (18.4% of the respondents 65 + compared to 6% of those 25−34 years old) and less educated than others (12.2% with secondary education compared to 6% of those with tertiary education), possibly suggesting that data are not missing completely at random (MCAR) but rather missing at random (MAR). These differences were statistically significant (χ²(5)=61.4, p = 0.000 and χ²(3)=27.5, p = 0.000 respectively). Demographic variables were much less affected (missingness range: 0.1%−1.8%). Since the missing values were mostly concentrated on the dependent variables, however, the impact on our estimates is likely to be modest. Finally, since the questionnaire was not formally validated, it remains possible that some questions were worded in ways that created confusion for participants, including across multiple languages. One example here is whether participants were clear that GGE involved heritable modifications or not.

## Conclusions

This study documents the views of the Swiss public towards SGE and GGE. It reveals moderate support for therapeutic SGE, with decreasing support as the conditions become less serious or move towards enhancement. Most reasons for hypothetically supporting gene editing treatments relate to views towards what it means to live a good life, and views towards science and medicine, rather than religious views. Our study findings map to those documented in other countries in some, but not all cases, and importantly show that even in a small country there can be significant variations in public views. This is important when developing country-specific and international policy regarding gene editing and other biomedical regulations. Results such as this study findings should inform future citizen consultations and policy development.

## Supporting information

S1 File Supplemental Methods: Includes operationalization of variables (Supplemental Table S1) and English survey measure. (PDF)

S2 File Supplemental Tables: Includes Supplementary Table 2 (S2): Participant demographics –regression analysis sample. Supplementary Table 3 (S3): Correlates of views on human gene editing (OLS Regression results). Supplementary Table 4 (S4): Correlates of views on potential uses of gene editing (OLS regression estimates).
(PDF)
