# Supplementary material for: Views of the Swiss public towards gene editing
Source: PLoS One. 2026 May 21;21(5):e0334305. doi: 10.1371/journal.pone.0334305 (PMC13193455; doi:10.1371/journal.pone.0334305)
Supplement: S1 File — (PDF) [file pone.0334305.s001.pdf]

SUPPLEMENTAL METHODS --

TABLE 1 – OPERATIONALIZATION OF VARIABLES

SURVEY (IN ENGLISH) – Non-English versions upon request from corresponding author (Kelly.ormond@hest.ethz.ch)

**Supplemental Methods Table S1: Operationalization of Variables**		
Variable name	Question	Code
Age group	Which age bracket do you fall into?	1 = 18-24, 2 = 25-34, 3 = 35-44, 4 = 45-54, 5 = 55-64, 6 = 65+
Gender	What is your gender?	1 = male, 2 = female, 3 = non-binary/fluid, 4 = Prefer not to say
Swiss Nationality		0 = Other Nationality, 1 = Swiss Nationality
Household size		Household size in persons: 1 = 1 person, 2 = 2 persons, 3 = 3-5 persons, 4 = 6+ persons
Marital status		1 = single, 2 = married, 3 = widowed, 4 = divorced, 6 = registered partnership, 7 = partnership
Tertiary education	Which is the highest educational level you have completed?	0 = Up to secondary or other corresponding education, 1 = tertiary education
Genetic condition	Do you or anyone in your family have an inherited or genetic condition?	0 = no, 1 = yes, 2 = unsure/don't know, 3 = Prefer not to say
Experience in medical or related fields	Have you ever worked (more than 1 year) or have a post-Matura qualification in one of the following areas? Medicine/medical studies, genetics, microbiology, biomedical sciences, biochemistry<br>and cell biology.	0 = no, 1 = yes,
Self-reported awareness	Which of the options below best describe your current awareness of human gene editing?	1 = I have never heard of it, 2 = Aware, limited knowledge, 3 = Knowledgeable, 4 = Unsure / don't know
Knowledge index	Please respond to the following statements as being either TRUE or FALSE based on your understanding of genetics and human gene editing. If you do not know the answer, please select<br>"Unsure/don't know".<br><br>1) If a genetic change is heritable, it can be passed on to future generations.	Additive scale 0-5 based on correct statements (0/1)

	2) Genes are sections of DNA that provide instructions for making proteins.<br><br>3) Mutations in DNA are always harmful.<br><br>4) Heritable human gene editing is currently permitted in Switzerland.<br><br>5) An embryo is created when an egg cell is fertilized by a sperm cell.	
Religious	Do you describe yourself as a religious person?	1 = not religious, 2 = somewhat religious, 3 = very religious
Voted in last election	Did you vote in the last federal election?	0 = no, 1 = yes, 2 = not eligible, 3 = prefer not to say

Supplemental Methods: Survey in English (*DE, FR and IT available on request*)

Swiss National Survey on Gene Editing: Formulating a Swiss Response

Welcome to our survey on gene editing!

Imagine that someone who had a medical illness or genetic condition could receive a treatment to change their genes and treat or cure them of the condition. This hypothetical treatment could happen to an already-alive person, or to a baby or fetus (during, or even before, a pregnancy). It could also hypothetically be done on an embryo (similar to the way that pre-implantation genetic testing is performed), or on eggs or sperm before conception happens. Currently human gene editing is restricted in Switzerland, but other countries are moving forward with some applications.

With this survey we want to know more about your attitude towards gene editing.
In particular, we are interested in whether and under which circumstances you would support the potential availability of gene editing in Switzerland. What concerns would you have? What would be your expectations? To get a better understanding of what is important to you, please answer the following questions.

The survey does not aim to test your knowledge about this topic, but rather to get your opinions and views. There are no right and wrong answers, and our research team is not promoting any particular outcome. It is only important that you answer the questions truthfully and to the best of your abilities.

How should I fill out the questionnaire?
§ You can fill it out on paper (here) or go online at www.ged.ethz.ch.
§ Tick the applicable box next to the selected answer.
§ If you accidentally check the wrong box, fill it in completely with a ballpoint pen and then check the applicable box.

Thank you very much for completing this survey!

Statement of Consent: By participating in the survey, you are giving your informed consent to participate in this research study.
Additional information about the interview study and your rights:
Right of withdrawal: You have the right to withdraw from the survey at any time. This can be done without specifying reasons and without any consequences. You can refuse to answer any question or stop the survey anytime without negative consequences for you.
Advantages and disadvantages for interviewees: There are no advantages or disadvantages associated with participating in the survey.
Data protection: The survey data will be stored safely in an encrypted form at ETH and reported in an anonymous way. Only the responsible investigators will have access to the original survey data, under strictly observed rules of confidentiality. De-identified quotations from the survey and summary data may be used for scientific publications or conference presentations.

First, a few questions about you

Password: ________________________

1. In which Kanton do you currently live?
    - **Aargau** (AG)
    - **Appenzell Innerrhoden** (AI)
    - **Appenzell Ausserrhoden** (AR)
    - **Basel-Land** (BL)
    - **Basel-Stadt** (BS)
    - **Bern / Berne** (BE)
    - **Freiburg / Fribourg** (FR)
    - **Geneve** (GE)
    - **Glarus** (GL)
    - **Graubünden** (GR)
    - **Jura** (JU)
    - **Luzern** (LU)
    - **Neuchâtel** (NE)
    - **Nidwalden (NW)**
    - **Obwalden (OW)**
    - **St Gallen** (SG)
    - **Schaffhausen** (SH)
    - **Schwyz** (SZ)
    - **Solothurn** (SO)
    - **Thurgau** (TG)
    - **Ticino** (TI)
    - **Uri** (UR)
    - **Vaud** (VD)
    - **Valais / Wallis** (VS)
    - **Zug** (ZG)
    - **Zürich** (ZH)

2. What is your gender?
    - Male
    - Female
    - Non-binary/fluid
    - Prefer not to say

3. Which age bracket do you fall into?
    - 18-24
    - 25-34
    - 35-44
    - 45-54
    - 55-64
    - 65+

4. Which is the highest educational level you have completed?
    - Obligatory school (9 years)
    - Basic vocational training/apprenticeship
    - High school degree (Matura)
    - Higher vocational training or technical school
    - University Bachelor's degree
    - University Graduate (Master or Doctoral) Diploma
    - Other, please specify in comment box

5. Have you ever worked (more than1 year), or have a post-Matura qualification in one of the following areas:
    - Medicine/medical studies
    - Genetics
    - Microbiology
    - Biomedical Sciences
    - Biochemistry and cell biology
    - None of the above

6. Do you describe yourself as a religious person?
    - Very much
    - Somewhat
    - Not at all

7. Did you vote in the last federal election?
    - Yes
    - No
    - No, I am not eligible to vote
    - Prefer not to say

8. Do you or anyone in your family have an inherited or genetic condition?
    - Yes
    - No
    - Unsure/don't know
    - Prefer not to say

Awareness of human gene editing

This part of the survey is meant to help us gauge your current awareness of gene editing. The questions are not intended to test your knowledge, but to make sure that we collect responses

from people with different amounts of awareness about the topic. It is not necessary to have any prior knowledge about gene editing to participate in the survey.

<u>Please do not go back and change any of these answers after completing the rest of the survey.</u>

Q1. Which of the options below best describe your current awareness of human gene editing?

- I have never heard of it.
- I have heard of it, but know little or nothing about it.
- I know enough about it that I could explain it to a friend.
- Unsure/don't know.

Q2. Please respond to the following statements as being either TRUE or FALSE based on your understanding of genetics and human gene editing. If you do not know the answer, please select "Unsure/don't know".

	True	False	Unsure/don't know
If a genetic change is heritable, it can be passed on to future generations.			
Genes are sections of DNA that provide instructions for making proteins.			
Mutations in DNA are always harmful.			
Heritable human gene editing is currently permitted in Switzerland.			
An embryo is created when an egg cell is fertilised by a sperm cell.			

About gene editing

Please read the following text which provides a brief introduction to gene editing. If you would like to know more about gene editing, we will link to further resources at the end of the survey.

Gene editing is a new development in science that allows targeted changes to be made to the DNA that all living things possess. These changes involve removing, adding, or exchanging sections of DNA (known as genes) that affect particular traits.

Some forms of gene editing are heritable, which means that the changes to the DNA are passed on to future generations. Sometimes you will see this called "Heritable Gene Editing" or "Germline Gene Editing". It is usually done on an embryo or on the eggs and sperm prior to conception. This is not currently allowed in Switzerland, even for research.

Other changes are non-heritable, which means they are not passed on and only affect the individual in which the changes are made. Sometimes you will see this called "Somatic Gene Editing" and it is done on a person that is already existing, generally to treat a rare inherited disease or illness. In Switzerland, it is estimated that nearly 500,000 people have a "rare disease". It could also be done during a pregnancy on an existing fetus.

So far, gene editing has mostly been used for research purposes. In some cases, the research is to better understand disease or to develop new drugs. Also, in countries outside of Switzerland, there are about 200 people who have had the non-heritable ("somatic") type of gene editing performed as part of a research study to treat a disease or illness that they have.

There are many possible practical applications that could happen in the future. Outside of humans, we are already using it to alter the genes of plants and animals to increase resistance to disease. In humans, it could also be used to prevent the inheritance of diseases in humans, and determine (and change) the attributes of babies and future people in ways that enhance them outside of medical uses ("enhancement"). These types of uses are not happening currently.

*** https://www.samw.ch/en/Projects/Overview-of-projects/Rare-diseases.html**

Views on human gene editing and its potential applications

The following questions are about your views on human gene editing and some of the ways it might be used.

Q3. Please read each of the following statement and respond how much you tend to disagree or agree with what you think it is communicating. There are no right and wrong answers – we are just interested in how you feel about each statement.

	Strongly disagree			Neutral			Strongly agree
	1	2	3	4	5	6	7
Parents have a right to edit the genes of their children before they are born.							
I'm concerned that gene editing will lead to a reduction in genetic diversity.							
I'm concerned that gene editing will be used as a quick techno-fix without actually dealing with real problems.							
Editing genes that will be inherited is problematic because it means making decisions for future people who don't exist yet.							
My cultural beliefs make me cautious about gene editing.							
When you change someone's genes you are fundamentally changing who they are.							

I worry that something will go wrong with gene technology.							
Gene editing technologies give us power over human life itself.							
We find strength when we face illness and adversity. It makes us who we are.							
	Strongly disagree			Neutral			Strongly agree
	1	2	3	4	5	6	7
If gene editing is proven to be safe, then I see no reason to oppose it.							
I would consider using gene editing for my children to give them an advantage in life, so long as it's safe.							
I would be OK with having my own genes edited.							
Each individual has a right to decide for themselves whether to undergo gene editing, as long as the changes cannot be passed on to future generations.							

If we are able to safely perform gene editing, we should make it available.							
Gene editing should be available only if there are no other treatment options available.							
If gene editing is available in other countries it should be available in Switzerland.							
Gene editing is eugenics.							
Gene editing will increase the inequities that already exist for people with rare disease and disability.							

Q4. How much do you agree or disagree with the following potential uses of human gene editing?

	1 Strongly disagree	2 Disagree	3 Neutral	4 Agree	5 Strongly agree
Editing the cells of children or adults to cure a life-threatening disease.					
Editing the cells of children or adults to cure a debilitating disease.					
Editing the cells of children or adults to cure a disease that led to minor physical impairments.					
Editing the cells of children or adults to cure a disease that led to significant learning impairments (for example, a person could not live independently) and also some moderate physical impairments.					
Editing the cells of children or adults to cure a disease that led to significant learning impairments but without any other medical impacts.					
Editing the cells of children or adults to cure a disease that started only in adulthood (for example, cancer or dementia).					
Editing the cells of children or adults to cure a disease that led to a sensory impairment, such as vision or hearing loss from birth.					

Editing the cells of children or adults to alter physical abilities—such as strength or sporting ability.					
Editing the cells of children or adults to alter cognitive abilities – such as memory or intelligence.					
Editing the cells of embryos to prevent a life-threatening disease.					
Editing the cells of embryos to prevent a debilitating disease.					
	Strongly disagree	Disagree	Neutral	Agree	Strongly agree
Editing the cells of embryos to alter physical abilities—such as strength or sporting ability.					
Editing the cells of embryos to alter cognitive abilities—such as memory or intelligence.					
Editing the cells of plants and animals used in food production.					
Editing human embryos in genome editing research.					

Q5. Are there any other uses of gene editing in humans (not listed above) that you would DISAGREE with? If so, which?

a. Yes
b. No
c. Unsure/don't know

Q6. Are there any other uses of gene editing in humans (not listed above) that you would AGREE with? If so, which?

a. Yes
b. No
c. Unsure/don't know

Q7**.** Are there any particular traits or illnesses in humans or non-human organisms that you would like to edit? If so please list and make a short statement as to why.

a. Yes
b. No
c. Unsure/don't know

Q8. We are also interested in understanding why you feel the way you do about human gene editing. Which of the following are factors that influence how you are feeling about human gene editing?

	1 Strongly makes me feel negatively	2 Slightly makes me feel negatively	3 Neutral	4 Slightly makes me feel positively	5 Strongly makes me feel positively
My own views towards religion.					
My own views towards science and biology.					
My own views towards what it					

means to be a parent/have children.					
My own views about illness and suffering.					
My own views towards what it means to "have a good life".					
My own views towards "what it means to be human" and what "life" means to me.					

Q8a. Please write a few short comments about the factors that have influenced your response or attitude toward human gene editing.

Attitudes towards the regulation of human gene editing

In 2024, we hope to bring together a group of ordinary Swiss persons in Zurich to participate in a Swiss Citizens' Jury on Gene Editing. This has already been done in several other countries across the world – Australia, France, UK – and many other events are planned. In each, the participants were ordinary members of the public – like yourselves – who were selected to represent the wider population of their country in terms of gender, age, education level and region. Over 3-4 days, the participants hear from experts, reflect, and discuss under what conditions they might accept the various forms of human gene editing. On the final day, the participants arrive at a set of policy recommendations, which are then shared with policy makers and regulators.

Q9. How much do feel that the following groups' voices should be heard in order to formulate good policies on gene editing?

	Not necessary						Completely necessary	Unsure/ don't know
	1	2	3	4	5	6	7	
Juries made up of ordinary citizens held across Switzerland.								
A Jury made up of people facing inherited diseases that was held in Switzerland.								
International organizations such as United Nations (esp WHO), the Organisation for Economic Co-operation and Development (OECD) or the European Council.								
A body of Swiss experts (scientists, doctors, lawyers, bioethicists).								

Q10. How important is it to you that the participants in a Swiss Citizen's Jury on Gene Editing:

	Not at all important 1	2	3	4	5	6	Very important 7	Unsure/ don't know
Are selected at random from the wider population.								
Are broadly reflective of the population.								
Engage in extensive small-group discussions.								
Listen to presentations from experts on gene editing.								
Listen to presentations from people who have lived experience with the types of conditions that might potentially be treated.								

Feedback and further information

Q11. Based on the information provided in this survey and/or your prior knowledge of gene editing, how confident did you feel expressing your views on human gene editing in this survey?

- Not confident at all
- Somewhat confident
- Very confident
- Unsure/don't know

Q12. If there is anything else that you would like to tell us about human gene editing, the research, or about the survey, please leave a comment in the box provided.

Thank you for completing our survey. Research like ours could not take place without people like you!

If you would be interested in being considered as a potential participant for the Swiss Citizen's Jury regarding gene editing in 2024, please go to https://bioethics.ethz.ch/research/GeneEditingEthic.html and enter your contact information. Any contact information on that page will not be linked to the survey data you have just entered.

If you would like to learn more about genome editing, we recommend the following resources:

In German:

- CRISPR-Cas einfach erklärt | Gentechnik | CrisprCas9 by BIOBYLUKE https://www.youtube.com/watch?v=4abtcn2pVzY
- CRISPR in 4 Minuten erklärt by youknow https://www.youtube.com/watch?v=gUa2H8CcUjU
- CRISPR-Cas9 – Biochemie / Molekularbiologie – AMBOSS Video by AMBOSS DE https://www.youtube.com/watch?v=R3WqGir9grQ
- CRISPR-Cas / CRISPR Cas9 [Ein Verfahren des Genom-editing] – [Biologie, Gentechnik, Oberstufe] by
- TeacherToby https://www.youtube.com/watch?v=6Fc4ajA7ft8

In French:

- CRISPR/CAS9 : une méthode révolutionnaire by Inserm https://www.youtube.com/watch?v=RplWR12npqM
- Comment fonctionnent les ciseaux moléculaires ? | Le tour de la question by La Croix https://www.youtube.com/watch?v=NBpKWMyWHE4
- Notre génome manipulé ? La révolution Crispr expliquée by Le Monde https://www.youtube.com/watch?v=4MiN_QNXS78&t=33s
- CRISPR Cas9 : Définitions, Principe et Applications | PRIX NOBEL DE CHIMIE 2020 by Biochimie Facile https://www.youtube.com/watch?v=lW64Y18tbUQ

In English:

- Genome Editing with CRISPR-Cas9 by McGovern Institute https://www.youtube.com/watch?v=2pp17E4E-O8
- How CRISPR lets you edit DNA – Andrea M. Henle by TED-Ed https://www.youtube.com/watch?v=6tw_JVz_IEc
- What is gene editing and how does it work? | The Royal Society by The Royal Society https://www.youtube.com/watch?v=XPDb8tqgfjY
- Gene Editing by ASGCT https://www.youtube.com/watch?v=i_1kLfK1cP0
- The Wellcome trust: https://wellcome.org/news/what-genome-editing-and-how-does-it-work
- The Australian Academy of Sciences: https://www.science.org.au/curious/crispr

Reports on genome editing from international and non-governmental agencies:

- European Commission and Directorate-General for Research and Innovation, *EGE Opinion on the Ethics of Genome Editing* (2021) https://op.europa.eu/publication/manifestation_identifier/PUB_KI0121062ENN
- World Health Organization, *Human Genome Editing: A Framework for Governance* (World Health Organization, 2021) https://apps.who.int/iris/handle/10665/342484
- Nuffield Council on Bioethics: https://www.nuffieldbioethics.org/publications/genome-editing-and-human-reproduction

US National Academies of Sciences, Engineering and Medicine: https://www.nap.edu/catalog/25665/heritable-human-genome-editing
