# Supplementary material for: Views of the Swiss public towards gene editing
Source: PLoS One. 2026 May 21;21(5):e0334305. doi: 10.1371/journal.pone.0334305 (PMC13193455; doi:10.1371/journal.pone.0334305)
Supplement: S2 File — Supplementary Table 3 (S3): Correlates of views on human gene editing (OLS Regression results). Supplementary Table 4 (S4): Correlates of views on potential uses of gene editing (OLS regression estimates). (PDF) [file pone.0334305.s002.pdf]

SUPPLEMENTAL TABLES

Supplementary Table 2: Participant demographics - regression analysis sample.

Supplementary Table 3: Correlates of views on human gene editing (OLS Regression results).

Supplementary Table 4: Correlates of views on potential uses of gene editing (OLS regression estimates)

Supplementary Table 2: Participant demographics - regression analysis sample

Variable	Number of people (N)	Unweighted percentage (%)	Weighted percentage (%)
**Total**	**3429**	**100**	**100**
**Language of survey completion**			
German	1497	43.7	66.2
English	153	4.5	5.6
Italian	1078	31.4	5.1
French	701	20.4	23.1
**Municipality language**			
German	1566	45.7	71.2
Italian	1119	32.6	4.2
French part	742	21.6	24.5
Rhaeto-Romanic	2	0.1	0.1
**Gender**			
Male	1673	48.8	50.0
Female	1711	49.9	48.8
Non-binary/fluid	13	0.4	0.4
Prefer not to say	32	0.9	0.8
**Age bracket**			
18-24	422	12.3	8.8
25-35	613	17.9	15.7
35-44	543	15.8	19.1
45-54	632	18.4	17.4
55-64	737	21.5	17.3
65+	482	14.1	21.7
**Nationality**			
Swiss	2748	80.1	82.0
Other	681	19.9	18.0
**Number of household members**			
1	499	14.6	14.5
2	1158	33.8	38.2
3-5	1711	49.9	45.4
6 or more	61	1.8	1.9
**Marital status**			
Single	1361	39.7	34.4

Married	1718	50.1	54.3
Widowed	54	1.6	1.8
Divorced	288	8.4	9.3
Registered partnership	6	0.2	0.2
Dissolved registered partnership	2	0.1	0.1
**Educational level**			
Up to Secondary education or Other	2421	70.6	72.0
Tertiary education (Bachelor's degree or higher)	1008	29.4	28.0
**Work experience in a related field (Medicine, Biology, Genetics etc.)**			
Yes	355	10.4	10.1
No	3074	89.7	89.9
**Self-reported awareness of gene editing**			
Never heard of gene editing	343	10.0	5.6
Aware, limited knowledge	2249	65.6	68.6
Knowledgeable	689	20.1	21.8
Unsure/don't know	148	4.3	3.9
**Self-reported level of religiosity**			
Very much	313	9.1	9.5
Somewhat	1530	44.6	42.9
Not at all	1586	46.3	47.6
**Participation in election**			
No	634	18.5	18.1
Yes	2018	58.9	61.0
No, not eligible to vote	587	17.1	16.0
Prefer not to say	190	5.5	4.8
**Genetic/inherited disease in family**			
No	1966	57.3	56.7
Yes	669	19.5	20.2
Unsure/don't know	730	21.3	21.7
Prefer not to say	64	1.9	1.5

Supplementary Table 3: Correlates of views on human gene editing (weighted and unweighted OLS Regression results)

Predictors of views on human gene editing (OLS Regression results)				
	(1a) DV: Support for gene editing (weighted)	(1b) DV: Support for gene editing (unweighted)	(2a) DV: Caution about gene editing (weighted)	(2b) DV: Caution about gene editing (unweighted)
Age group (ref. 18-24)				
25 – 34	-0.146	-0.208	-0.020	0.034
	(0.142)	(0.120)	(0.130)	(0.114)
35 – 44	-0.230	-0.322*	0.112	0.186
	(0.154)	(0.135)	(0.152)	(0.129)
45 – 54	-0.258	-0.317*	0.233	0.298*
	(0.161)	(0.137)	(0.155)	(0.130)
55 – 64	-0.168	-0.279*	0.135	0.212
	(0.161)	(0.141)	(0.162)	(0.134)
65+	-0.136	-0.174	0.213	0.341*
	(0.189)	(0.160)	(0.193)	(0.152)
Gender (ref. Male)				
Female	-0.452***	-0.324***	0.334***	0.295***
	(0.077)	(0.063)	(0.077)	(0.060)
Non-binary/fluid	-0.740	-0.827	-0.772	-0.331
	(0.507)	(0.498)	(0.869)	(0.474)
Prefers not to say	-0.758	-0.527	0.448	0.261
	(0.468)	(0.325)	(0.342)	(0.309)
Swiss	-0.265	-0.382*	0.015	0.157
	(0.189)	(0.168)	(0.195)	(0.160)
Number of household members (ref. 1)				
2	0.295*	0.248*	-0.191	-0.117
	(0.131)	(0.103)	(0.125)	(0.098)
3-5	0.186	0.199	-0.195	-0.171
	(0.128)	(0.105)	(0.125)	(0.099)
6 persons or more	0.053	-0.038	-0.263	-0.050
	(0.265)	(0.249)	(0.311)	(0.236)

Marital status (ref. Single)				
Married	-0.036	-0.035	0.030	0.012
	(0.113)	(0.094)	(0.112)	(0.089)
Widowed	-0.076	-0.117	-0.071	-0.071
	(0.336)	(0.259)	(0.348)	(0.246)
Divorced	-0.036	-0.028	0.203	0.170
	(0.167)	(0.133)	(0.167)	(0.127)
Registered partnership	1.468**	1.167	-0.430	-1.164
	(0.483)	(0.732)	(1.067)	(0.696)
Dissolved partnership	0.320*	0.236	-0.183	0.794
	(0.157)	(1.260)	(0.270)	(1.199)
Tertiary education	0.199*	0.127	-0.426***	-0.352***
	(0.093)	(0.075)	(0.096)	(0.071)
Genetic or inherited condition (ref. No)				
Family member with genetic condition	0.516***	0.487***	-0.025	0.018
	(0.101)	(0.081)	(0.101)	(0.077)
Unsure / don't know	0.117	0.156*	0.102	0.102
	(0.094)	(0.078)	(0.090)	(0.075)
Prefer not to say	-0.511	-0.441	0.887**	0.526*
	(0.325)	(0.242)	(0.324)	(0.230)
Experience in medical or related fields	0.088	-0.017	-0.276*	-0.222*
	(0.135)	(0.104)	(0.135)	(0.099)
Self-reported awareness (ref. Never heard of gene editing)				
Aware, limited knowledge	0.504**	0.425***	-0.074	-0.081
	(0.166)	(0.111)	(0.168)	(0.106)
Knowledgeable	0.682***	0.541***	-0.360	-0.304*
	(0.194)	(0.133)	(0.194)	(0.126)
Unsure / don't know	0.293	0.082	-0.677**	-0.463**
	(0.207)	(0.178)	(0.257)	(0.170)
Knowledge index	0.002	0.044	-0.056	-0.065*

	(0.034)	(0.027)	(0.036)	(0.026)
Religious (ref. Not at all)				
Somewhat religious	-0.120	-0.201**	0.315***	0.379***
	(0.082)	(0.066)	(0.082)	(0.062)
Very religious	-0.845***	-0.944***	0.758***	0.813***
	(0.143)	(0.112)	(0.150)	(0.107)
Voted in last election (ref. No)				
Yes	-0.175	-0.100	0.008	0.021
	(0.110)	(0.086)	(0.096)	(0.082)
Not eligible	0.046	-0.069	-0.206	-0.066
	(0.193)	(0.173)	(0.204)	(0.165)
Prefer not to say	-0.071	-0.103	-0.037	-0.115
	(0.182)	(0.153)	(0.181)	(0.145)
Language of survey completion (ref. German)				
English	0.332	0.450**	0.238	0.209
	(0.187)	(0.162)	(0.186)	(0.154)
French	-0.185*	-0.154	0.266**	0.278***
	(0.089)	(0.084)	(0.087)	(0.080)
Italian	0.757***	0.726***	0.070	0.079
	(0.095)	(0.076)	(0.103)	(0.072)
_cons	3.799	3.885	7.317	7.065
	(0.289)	(0.243)	(0.304)	(0.231)
N	3429	3429	3429	3429

Supplementary Table 4: Correlates of views on potential uses of gene editing (OLS regression estimates)

Predictors of views on potential uses of gene editing (OLS regression estimates)				
	(3a) DV: Views on therapeutic gene editing	(3b) DV: Views on therapeutic gene editing	(4a) DV: Views on gene editing for enhancement	(4b) DV: Views on gene editing for enhancement (unweighted)
Age group (ref. 18-24)				
25 – 34	-0.055	-0.085	-0.039	0.016
	(0.086)	(0.074)	(0.081)	(0.065)
35 – 44	-0.148	-0.169*	-0.002	-0.057
	(0.097)	(0.084)	(0.085)	(0.073)
45 – 54	-0.222*	-0.218**	-0.103	-0.083
	(0.101)	(0.084)	(0.085)	(0.074)
55 – 64	-0.036	-0.131	-0.007	-0.038
	(0.099)	(0.087)	(0.089)	(0.076)
65+	0.010	-0.070	0.016	-0.075
	(0.116)	(0.098)	(0.101)	(0.086)
Gender (ref. Male)				
Female	-0.260***	-0.190***	-0.306***	-0.270***
	(0.049)	(0.038)	(0.039)	(0.034)
Non-binary/fluid	-0.661	-0.394	-0.506*	-0.407
	(0.439)	(0.296)	(0.233)	(0.260)
Prefers not to say	-0.778*	-0.526**	-0.081	-0.062
	(0.315)	(0.201)	(0.269)	(0.177)
Swiss	-0.164	-0.218*	-0.344**	-0.282**
	(0.116)	(0.103)	(0.127)	(0.090)

Number of household members (ref. 1)				
2	0.232**	0.167**	0.046	0.050
	(0.080)	(0.063)	(0.064)	(0.055)
3-5	0.232**	0.189**	0.037	0.049
	(0.077)	(0.064)	(0.068)	(0.056)
6 persons or more	0.044	-0.058	0.058	0.047
	(0.193)	(0.156)	(0.188)	(0.137)
Marital status (ref. Single)				
Married	-0.131	-0.093	-0.125*	-0.126*
	(0.071)	(0.057)	(0.059)	(0.050)
Widowed	-0.059	-0.023	-0.258*	-0.195
	(0.172)	(0.156)	(0.124)	(0.137)
Divorced	-0.092	-0.100	-0.137	-0.086
	(0.104)	(0.081)	(0.079)	(0.071)
Registered partnership	0.624*	0.745	1.118**	1.087**
	(0.251)	(0.451)	(0.380)	(0.396)
Dissolved partnership	-0.131	-0.093	-0.125*	-0.126*
	(0.071)	(0.057)	(0.059)	(0.050)
Tertiary education	0.255***	0.198***	0.041	-0.055
	(0.059)	(0.045)	(0.047)	(0.040)
Genetic or inherited condition (ref. No)				
Family member with genetic condition	0.201***	0.162**	0.029	0.024
	(0.059)	(0.049)	(0.052)	(0.043)

Unsure / don't know	0.086	0.100*	0.022	0.047
	(0.059)	(0.048)	(0.049)	(0.042)
Prefer not to say	-0.535**	-0.487***	-0.270	-0.094
	(0.188)	(0.144)	(0.160)	(0.126)
Experience in medical or related fields	0.127	0.027	0.022	0.006
	(0.080)	(0.064)	(0.063)	(0.056)
Self-reported awareness (ref. Never heard of gene editing)				
Aware, limited knowledge	0.207*	0.175**	-0.110	-0.138*
	(0.098)	(0.068)	(0.090)	(0.059)
Knowledgeable	0.203	0.142	-0.019	-0.043
	(0.116)	(0.081)	(0.101)	(0.071)
Unsure / don't know	0.084	-0.049	0.344*	0.179
	(0.140)	(0.113)	(0.139)	(0.099)
Knowledge index	0.050*	0.069***	-0.098***	-0.100***
	(0.021)	(0.016)	(0.018)	(0.014)
Religious (ref. Not at all)				
Somewhat religious	-0.060	-0.058	-0.080	-0.047
	(0.051)	(0.040)	(0.040)	(0.035)
Very religious	-0.500***	-0.492***	-0.047	-0.094
	(0.097)	(0.069)	(0.069)	(0.060)
Voted in last election (ref. No)				
Yes	-0.111	-0.064	-0.167**	-0.118*
	(0.069)	(0.052)	(0.057)	(0.046)

Not eligible	0.092	0.009	-0.130	-0.097
	(0.115)	(0.106)	(0.132)	(0.093)
Prefer not to say	-0.015	-0.010	-0.005	-0.008
	(0.110)	(0.094)	(0.100)	(0.083)
Language of survey completion (ref. German)				
English	0.057	0.164	0.342**	0.507***
	(0.114)	(0.100)	(0.112)	(0.088)
French	0.137*	0.164**	0.009	0.031
	(0.054)	(0.051)	(0.046)	(0.045)
Italian	0.298***	0.300***	0.198***	0.265***
	(0.060)	(0.046)	(0.057)	(0.041)
_cons	3.881***	3.923***	3.049***	2.981***
	(0.173)	(0.148)	(0.175)	(0.130)
N	3490	3490	3490	3490
